# Joint Position and General Hypermobility Affect Elbow Joint Congruence on Magnetic Resonance Imaging: A Prospective Cohort Study

**DOI:** 10.1177/03635465251330152

**Published:** 2025-04-15

**Authors:** Stephanie Geyer, Maximilian Hinz, Pavel Kadantsev, Sebastian Lappen, Philipp W. Winkler, Jan Neumann, Benedikt J. Schwaiger, Sebastian Siebenlist

**Affiliations:** * Department of Sports Orthopaedics, Technical University of Munich, Munich, Germany; † Department of Shoulder and Elbow Surgery, St Vinzenz Klinik, Pfronten, Germany; ‡ Center for Musculoskeletal Surgery, Charité, Berlin, Germany; § Department of Orthopaedic and Trauma Surgery, University Medical Centre, Mannheim, Medical Faculty Mannheim, University of Heidelberg, Mannheim, Germany; ‖ Department of Orthopaedics and Traumatology, Kepler University Hospital GmbH, Johannes Kepler University Linz, Linz, Austria; ¶ Department of Radiology, Kantonsspital Graubünden, Chur, Switzerland; # Department of Diagnostic and Interventional Neuroradiology, Technical University of Munich, Munich, Germany; Investigation performed at the Department of Sports Orthopaedics, Technical University of Munich, Munich, Germany

**Keywords:** hypermobility, elbow, joint incongruence, general laxity, MRI, posterolateral instability

## Abstract

**Background:**

Patients with posterolateral rotational instability (PLRI) of the elbow exhibit a higher degree of joint incongruence on magnetic resonance imaging (MRI) than patients without PLRI. However, the influence of joint hypermobility and position of the elbow in healthy participants is yet unknown.

**Purpose:**

To analyze the influence of general hypermobility and elbow joint position on joint congruence in healthy participants via MRI.

**Study Design:**

Case-control study; Level of evidence, 4.

**Methods:**

Twenty participants with Beighton score <5 (normal group) and 20 with Beighton score ≥5 (hypermobile group) who had healthy elbows underwent bilateral MRI in full extension and supination, full extension and pronation, and 30° of flexion in both supination and pronation. Sagittal radiocapitellar joint incongruence and ulnohumeral joint incongruence (in sagittal, coronal, and axial planes) were measured according to a standardized protocol.

**Results:**

Radiocapitellar congruence increased in pronation (*P* < .001) but did not change between flexion and extension (*P* > .05). Coronal ulnohumeral congruence increased significantly from extension and supination to pronation (*P* = .010) and to combined flexion and pronation (*P* = .011). Sagittal and axial ulnohumeral joint congruence did not change significantly between different elbow joint positions (*P* > .05). Significant differences between the normal and hypermobile groups were observed in 3 of the 4 evaluated joint positions. Overall, the hypermobile group showed an increased joint incongruence except in extension and pronation (*P* > .05).

**Conclusion:**

In MRI examination of healthy elbows, joint congruence increased significantly from supination to pronation. In combined extension and pronation, the elbow joint was equally congruent in the normal and hypermobile groups. When MRI scans in supination or combined flexion and pronation are evaluated, increased joint incongruence in hypermobile individuals is physiological and should not be confused with PLRI.

The diagnosis of elbow instability remains challenging, especially in chronic cases when hypermobility or stiffness may be present.^7^ Magnetic resonance imaging (MRI) provides sufficient information about the integrity of the ligaments and severity of soft tissue lesions in acute injuries but is insufficient in patients with chronic elbow instability.^5,19^ In these cases, attention should be paid to the bony configuration, such as a wide trochlear notch, as this may predispose patients to elbow instability.^19^

Elbow joint incongruence on MRI can also be considered as a sign of instability.^8^ Hackl et al^8^ showed that patients with a clinically confirmed diagnosis of posterolateral rotatory instability (PLRI) had an increased joint incongruence on MRI scan compared with a group of patients with chronic lateral elbow pain and a negative posterior drawer test as well as a negative pivot-shift test. Kim et al^12^ demonstrated that posterior translation of the radial head on sagittal MRI scans correlated with pathological changes in lateral elbow stabilizers in patients with chronic radial epicondylopathy, including the extensor muscles. However, neither study included healthy controls as a comparison group, which may limit the extrapolation of their results.

To date, the degree of physiological incongruence in healthy elbows on MRI scans is not known. Furthermore, it is not understood how elbow joint incongruence is affected by the elbow joint position or the presence of general hypermobility. Therefore, the aim of this study was to investigate whether joint position and general hypermobility influenced elbow joint congruence on MRI in a population with healthy elbows. To the best of our literature research, this is the first comparative survey that includes MRI examination in healthy individuals to assess hypermobility in elbow joints.

It was hypothesized that elbows would be more congruent in pronation, as the bony configuration may provide more stability in this position. In addition, it was hypothesized that participants in the hypermobile group would have an overall greater joint incongruence compared with participants in the normal group.

## Methods

The presented study was approved by the ethics committee of the Technical University of Munich (reference 552/19 S-KH) and conducted according to the Declaration of Helsinki. All participants signed written informed consent forms.

Twenty participants (10 male and 10 female) with and 20 participants (10 male and 10 female) without general joint hypermobility who had healthy elbow joints prospectively underwent MRI of both elbows (80 elbows joints in total). We excluded participants with elbow complaints in the past 6 months, a medical history of epicondylopathy, radial head or elbow dislocation, elbow or forearm fractures, or elbow, forearm, or hand surgery and those with connective tissue disorders or other relevant diseases. General joint hypermobility was assessed before enrollment and confirmed by the first author (S.G.) on the day of the MRI using the Beighton score,^2^ with a score ≥5 indicating general joint hypermobility.

### Magnetic Resonance Imaging

MRI (3D, proton density weighted, spectral attenuated inversion recovery [SPAIR], sagittal acquisition) was performed in the same machine using a standardized protocol with 4 different standardized goniometer-controlled positions: (1) full elbow extension and supination, (2) full elbow extension and pronation, (3) 30° of elbow flexion and supination, and (4) 30° of elbow flexion and pronation. The test participants were placed in a supine position with their arm outstretched next to their body. The goniometer was placed at the marked humeroradial joint line for measurements. For position 1, the arm was passively brought into full extension and was controlled by the goniometer at 0° of flexion. Supination was actively performed by the participant until the back of the thumb tip reached the MRI tube ground and was then weighted down with a sandbag. In position 2, the extension was controlled in the same way and the pronation was actively achieved by placing the flat hand on the base of the MRI tube. No weight was necessary to secure the pronated position because it is more physiological. A 30° customized support wedge and Velcro tape were used to achieve a standardized flexion angle of 30°, and the angle was controlled by a goniometer. Supination (position 3) and pronation (position 4) in the flexion position were aligned on the plane wedge. Before each MRI examination, the corresponding positions and angles were checked again.

### Evaluation of Elbow Joint Congruence

**Figure 1.**
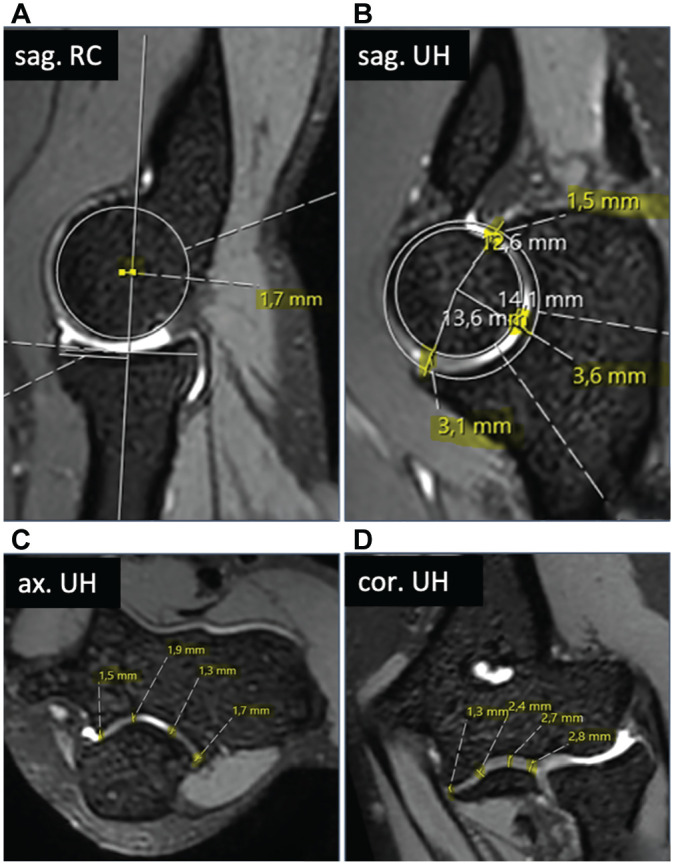
Standardized measurements of joint congruence: (A) radiocapitellar incongruence in the sagittal plane; ulnohumeral incongruence in the (B) sagittal, (C) axial, and (D) coronal planes.

Joint congruence, via radiocapitellar congruence in the sagittal plane and ulnohumeral congruence in the sagittal, axial, and coronal planes, was assessed by 2 orthopaedic surgeons (S.G., P.K.) using the previously established measurement method from Hackl et al^8^ (Figure 1). The measurements were repeated after 2 weeks by 1 orthopaedic surgeon (P.K.). Negative values for radiocapitellar distance mean that the radial head is ventral to the center of the capitellum. The axial plane was defined by aligning the sagittal and coronal planes along the long axis of the humeral shaft in the coronal and sagittal planes, respectively. After all planes were defined, the first image showed the sagittal view of the center of the radial head. The position of the center was confirmed using the 2-circle method, and the midline of the radial head before the longitudinal axis of the proximal radius was drawn. The long axis of the proximal radius and the center of rotation of the capitulum were marked and the radiocapitellar distance was measured (Figure 1A: radiocapitellar incongruence). For the second measurement in the sagittal plane, the coronoid process was superimposed, 2 circles were drawn on the articular surface of the olecranon and the trochlea, and the distances between the 2 circles at the olecranon, at the tip of the coronoid process, and between the 2 predefined points were measured (Figure 1B: sagittal ulnohumeral incongruence). The axial ulnohumeral measurements were taken through the axis of motion of the distal humerus at the radial and ulnar edges and at 2 points between the measurements (Figure 1C: axial ulnohumeral incongruence). In the coronal view, the ulnohumeral distance was measured at 4 points: at the ulnar and radial edges of the ulnohumeral joint and at 2 points in the middle of the ulnohumeral joint (Figure 1D: coronal ulnohumeral incongruence). For the sagittal, axial, and coronal planes, the mean value was calculated from the 4 measurements and designated as the respective distance.

All measurements were conducted using the image analysis system Philips iSite PACS (Koninklijke Philips), which allows for a multiplanar reconstruction. The measurements were carried out randomly, regardless of group affiliation (normal vs hypermobile).

### Statistical Analysis

A priori sample size calculation was based on a previous study evaluating PLRI of the elbow using MRI.^8^ A mean radiocapitellar incongruence of 2.9 ± 2.7 mm and 1.3 ± 1.0 mm was assumed for unstable (ie, hypermobile) and stable (ie, normal) elbows, respectively.^8^ Consequently, a minimum of 54 elbows (27 elbows per group) were required to achieve a statistical power of 0.80 (effect size, 0.79).

Data were analyzed using SPSS 28.0 (IBM-SPSS). Categorical variables are presented as numbers. Normal distribution of continuous variables was assessed using the Shapiro-Wilk test and graphically confirmed. Nonnormally distributed continuous variables are shown as median (25%-75% interquartile range [IQR]). For group comparisons of continuous variables, an unpaired *t* test (normally distributed data) or Mann-Whitney *U* test (nonnormally distributed data) was applied.

Intra- and interrater reliabilities of all measurements were assessed via the intraclass correlation coefficient (ICC) and interpreted according to Cichetti^4^ (<0.40, poor; 0.40-0.59, moderate; 0.60-0.74, good; ≥0.75, excellent). Means of the 2 raters were used for presentation of the measurements in the Results section.

## Results

In total, 40 participants with healthy elbows were included in the study. Both groups (normal and hypermobile) consisted of 10 male and 10 female participants. The Beighton score was significantly higher in the hypermobile group than in the normal group (median 7.5 [IQR 6.0-9.0] vs median 0 [IQR 0-2.0], respectively; *P* < .001). No significant difference with regard to age was found between groups (hypermobile, median 31.0 years [IQR 27.0-37.3 years]; normal, median 28.5 years [IQR 27.0-31.0 years]; *P* = .500).

### Influence of Joint Position on Elbow Joint Congruence

**Figure 2.**
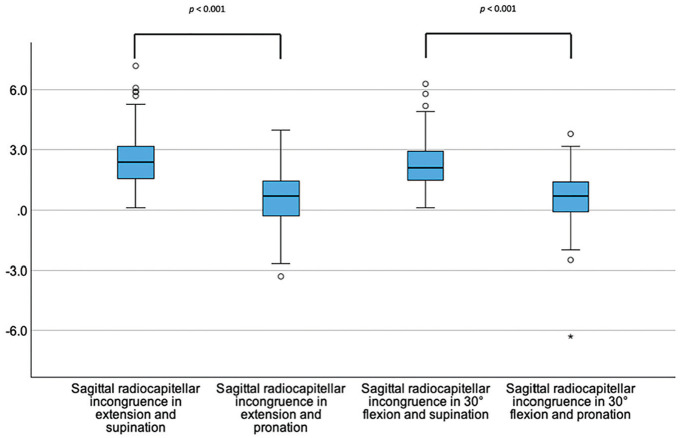
Box plots showing the sagittal radiocapitellar joint incongruence (y-axis, in mm) dependent on the position of the elbow joint.The asterisk (*) marks an extreme value. The circle (○) represents an outlier.

Sagittal radiocapitellar congruence increased in pronation (*P* < .001) but did not change with flexion vs extension (*P* > .05) (Figure 2).

**Figure 3.**
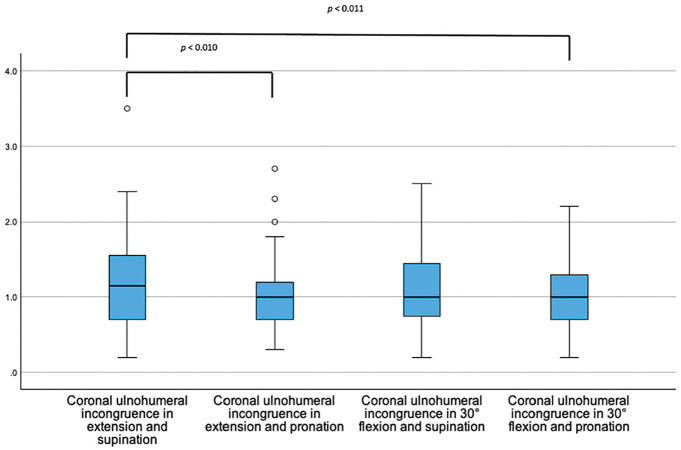
Box plots showing the coronal ulnohumeral joint incongruence (y-axis, in mm) dependent on the position of the elbow joint.

Coronal ulnohumeral congruence increased significantly in pronation (*P* = .010) and combined flexion and pronation (*P* = .011) when compared with extension and supination (Figure 3).

**Figure 4.**
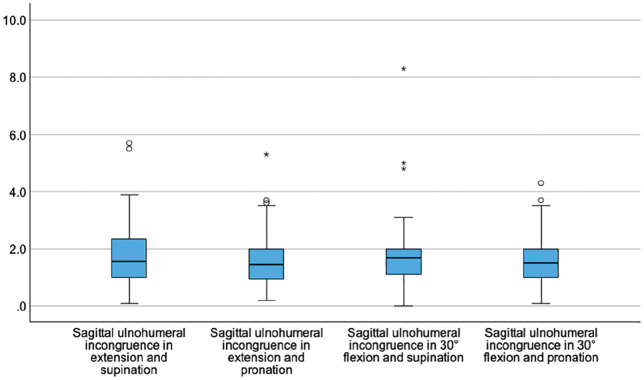
Box plots showing the sagittal ulnohumeral joint incongruence (y-axis, in mm) dependent on the position of the elbow joint.

**Figure 5.**
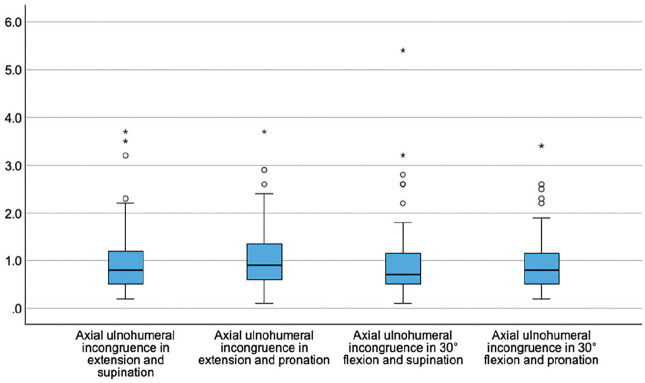
Box plots showing the axial ulnohumeral joint incongruence (y-axis, in mm) dependent on the position of the elbow joint.

Sagittal and axial ulnohumeral joint congruence did not change significantly with elbow flexion or forearm rotation (*P* > .05) (Figures 4 and 5).

### Influence of Hypermobility on Elbow Joint Congruence and Joint Position

**Figure 6.**
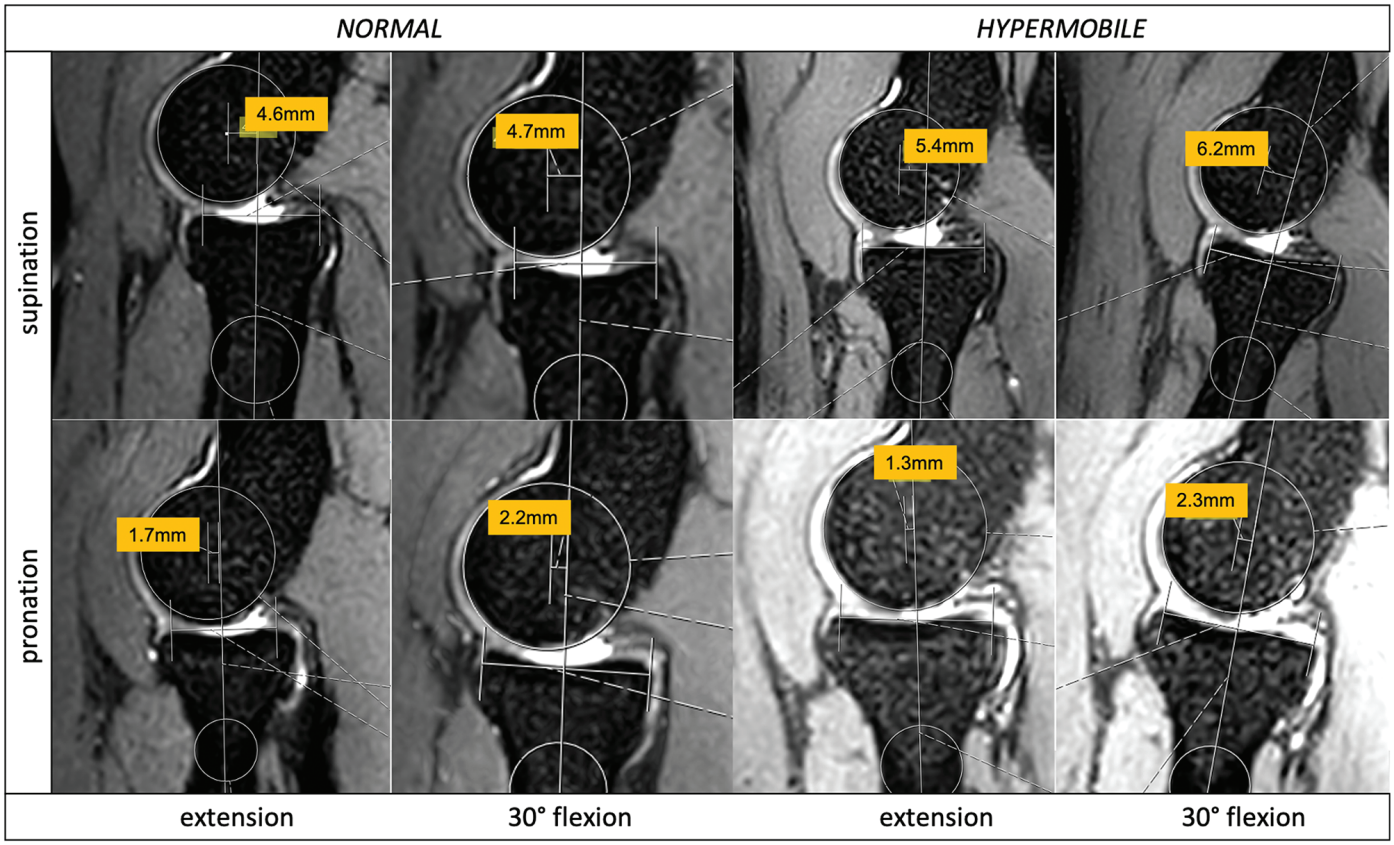
Comparative views for measurements of 2 participants (normal vs hypermobile) showing sagittal radiocapitellar joint incongruence (in millimeters) dependent on the position (y-axis, pronation and supination; x-axis, extension and flexion).

Differences in elbow joint congruence were observed between the normal and hypermobile groups in extension and supination, 30° of flexion and supination, and 30° of flexion and pronation (Figure 6).

**Figure 7.**
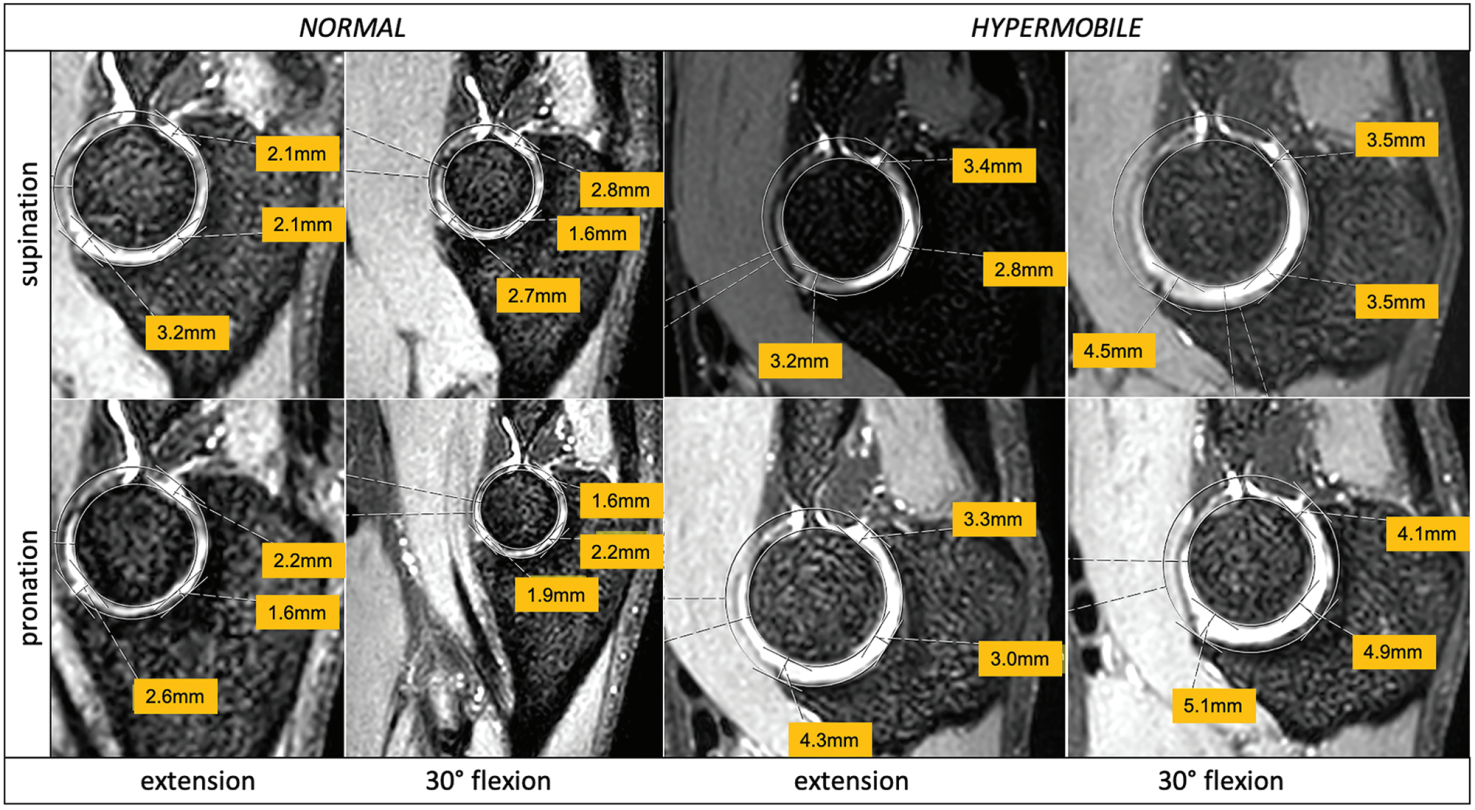
Comparative views for measurements of 2 participants (normal vs hypermobile) showing sagittal ulnohumeral joint incongruence (in millimeters) dependent on the position (y-axis, pronation and supination; x-axis, extension and flexion).

In extension and supination, coronal ulnohumeral congruence was significantly higher in the normal group (*P* = .004). In contrast, in 30° of flexion and supination, sagittal radiocapitellar congruence was higher in the hypermobile group (*P* = .039), but sagittal ulnohumeral congruence was lower in the hypermobile group (*P* = .002) (Figure 7).

In 30° of flexion and pronation, coronal ulnohumeral joint congruence was significantly higher in the normal group (*P* = .009). Only in extension and pronation did elbow joint congruence not differ significantly between groups (Table 1).

**Table 1:** Comparison of Joint Incongruence of Normal vs Hypermobile Elbows in Different Joint Positions^*a*^

Joint Position, MRI Plane, Incongruence	Joint Incongruence, mm		*P*
	Normal	Hypermobile	
Extension and supination			
Sagittal—radiocapitellar incongruence	2.4 (1.5-3.2)	2.5 (1.6-3.3)	.973
Sagittal—ulnohumeral incongruence	1.5 (0.9-2.0)	1.9 (1.0-2.5)	.252
Coronal—ulnohumeral incongruence	0.9 (0.6-1.5)	1.4 (0.9-1.7)	.**004**
Axial—ulnohumeral incongruence	0.6 (0.5-1.1)	0.9 (0.5-1.3)	.222
Extension + pronation			
Sagittal—radiocapitellar incongruence	0.7 (0.3-1.4)	0.7 (0.4-1.7)	.828
Sagittal—ulnohumeral incongruence	1.5 (1.0-1.9)	1.5 (0.7-2.5)	.637
Coronal—ulnohumeral incongruence	0.9 (0.7-1.2)	1.1 (0.7-1.4)	.445
Axial—ulnohumeral incongruence	0.9 (0.6-1.4)	0.9 (0.5-1.4)	.942
30° of flexion + supination			
Sagittal—radiocapitellar incongruence	2.5 (1.8-3.3)	1.9 (1.2-2.6)	.**039**
Sagittal—ulnohumeral incongruence	1.3 (0.8-1.8)	1.9 (1.4-2.1)	.**002**
Coronal—ulnohumeral incongruence	0.9 (0.7-1.2)	1.2 (0.8-1.6)	.067
Axial—ulnohumeral incongruence	0.7 (0.5-1.2)	0.8 (0.5-1.2)	.950
30° of flexion + pronation			
Sagittal—radiocapitellar incongruence	0.9 (0.1-1.4)	0.6 (0.4-1.4)	.464
Sagittal—ulnohumeral incongruence	1.5 (0.9-1.9)	1.5 (1.1-2.1)	.418
Coronal—ulnohumeral incongruence	0.9 (0.5-1.1)	1.2 (0.7-1.6)	.**009**
Axial—ulnohumeral incongruence	0.8 (0.5-1.1)	0.8 (0.5-1.2)	.502

a Data presented as median (25%-75% interquartile range); significant *P* values are bolded.

### Reliability of Measurements

Intrarater reliability was “excellent” (ICC = 0.788; 95% CI, 0.788-0.818). Interrater reliability was “moderate” (ICC = 0.433; 95% CI, 0.326-0.519).

## Discussion

The most important finding of the present study was that there was an association between different elbow joint positions and sagittal radiocapitellar and coronal ulnohumeral joint congruence. Overall, the hypothesis that joint congruence increases significantly with pronation compared with supination was confirmed. In addition, significant differences in joint congruence were observed between participants in the normal and hypermobile groups. Only in extension and pronation was the elbow joint equally congruent in the normal and hypermobile groups. In supination and in combined flexion and pronation, an increased joint incongruence was measured in hypermobile participants, which may be considered physiological and must not be misinterpreted as PLRI.

MRI is a noninvasive and user-independent imaging modality. Using MRI, Hackl et al^8^ showed that a significantly greater sagittal radiocapitellar and axial ulnohumeral joint incongruence was present in patients with PLRI. The results of the present study showed that the median sagittal radiocapitellar incongruence in healthy elbows was greater than the threshold value of 2.0 mm recommended by Hackl et al to identify patients with PLRI. In contrast, median axial ulnohumeral incongruence was lower in the normal and hypermobile cohorts than the threshold of 1.0 mm recommended by Hackl et al. The findings from the present study in a cohort with healthy elbows suggest that the MRI reference values for sagittal radiocapitellar incongruence may be adjusted upward to confirm PLRI, especially in the presence of general hypermobility. It is also worth noting that all MRI examinations in the study by Hackl et al were performed in extension and supination, which led to a limited applicability to MRI examinations that are not performed in extension and supination. Although the elbow is a congruent joint due to its 3-joint nature, the congruence of the elbow changed depending on the joint position in which MRI was performed. Joint incongruence of the radiocapitellar joint is higher in supination than in pronation due to the ellipsoid shape of the radial head.^14^ When elbow position changes from supination to pronation, a proximalization of the radial head is also noticed. This leads to an increase in the contact surface in the radiocapitellar joint and, thus, to an increase in joint congruence.^14^ Radiocapitellar congruence may increase further via axial loading^3^ or activation of lateral elbow stabilizers.^12^ In a 3-dimensional in vivo computed tomography (CT) study by Omori et al,^14^ the congruence of the ulnohumeral joint was less affected by forearm rotation. Previous studies, however, reported a valgus shift from supination to pronation of 7.1° in the coronal and axial plane on MRI, which contributes to the congruence of the joint, associated with contraction of the anconeus muscle.^11,16^ These results are in partial agreement with the present study, which showed a significant increase in coronal ulnohumeral congruence during pronation and combined flexion and pronation but no change in axial or sagittal ulnohumeral congruence during elbow flexion or forearm rotation. In addition, the arm is more often pronated than supinated during daily activities.^9^ Further, prone positioning of the patient with the elbow in pronation may be more comfortable than the “superman position” (patient supine, arms extended and elbows supinated).

The presence of hypermobility has become increasingly important in recent years, both diagnostically and in the treatment of joint instability.^10,17,18^ However, elbow findings associated with general hypermobility are much less clear in the literature. An association between hypermobility and PLRI has been suggested.^6^ To differentiate clinically between normal and hypermobile groups, the Beighton score is usually used.^2^ To date, MRI of the elbow joint has not helped to differentiate between these cohorts. However, this examination could be used in the future to establish standard values for normal and hypermobile individuals, especially in differentiating between hypermobile individuals and patients with PLRI. Currently, the data available to differentiate between healthy normal and hypermobile elbows and those with PLRI are limited.^1,8,13,15^ Kirschbaum et al^13^ compared the joint congruence in healthy elbows (both normal and hyperlax) and unstable elbows using ultrasound. Although a significant difference was found between normal and unstable elbows and between hypermobile and unstable elbows, no difference was found between normal and hypermobile elbows. The interrater reliability was excellent (ICC = 0.82) for the radial measurements, which is crucial for an accurate diagnosis of PLRI. Plath et al^15^ used ultrasound examination in addition to the arthroscopic rod method to diagnose the presence of PLRI in cadavers. The arthroscopic rod method showed excellent intra- and interrater reliability, whereas ultrasound examination showed only good intrarater reliability and poor interrater reliability, indicating that the arthroscopic rod method is an excellent tool for diagnosing PLRI in patients undergoing elbow surgery. Due to its invasiveness, however, its feasibility is limited. Arrigoni et al^1^ described arthroscopic intra-articular signs of “minor instability” of the elbow, such as ballottement of the radial head and, intra-articularly, synovitis and lateral capitellar chondropathy. Zagarella et al^20^ published a semiquantitative index for these signs of minor instability using CT arthrography with good reproducibility reported. However, due to the radiation exposure, CT examinations are suitable only for certain indications and therefore may not be used as extensively as MRI. In addition to the invasiveness of the examination method, no distinction was made between hypermobile patients and those with instability in the presence of chronic lateral elbow pain.^20^

In the current study, significant differences in elbow joint congruence were found between participants in the normal and hypermobile groups in all positions except extension and pronation. Because the effect of hypermobility on joint congruence may be eliminated on MRI in extension and pronation, it can be assumed that a false-positive diagnosis of PLRI could be avoided if MRI would be performed routinely in extension and pronation.

### Limitations

Some limitations should be considered when interpreting the study’s results. The threshold value of 5 for the Beighton score was potentially too low to extrapolate the results to patients with severe hypermobility. Because significant differences were already found at low levels of hypermobility, however, it may be assumed that this effect is amplified in participants with a greater degree of general laxity. Furthermore, transferability of the results of the present study to everyday clinical practice is limited because the interrater reliability was only moderate. Some of the differences were as small as 0.1 mm, and the joint surface was not imaged as accurately as would have been possible using CT. Furthermore, it is not known how axial loading of the joint or muscle strength and tension affect joint congruence and whether this would conceal signs of hypermobility.

## Conclusion

Joint congruence of healthy elbows on MRI increased significantly when changing from supination to pronation. In combined extension and pronation, the elbow joint was equally congruent in participants in the normal and hypermobile groups. When MRI scans in supination or combined flexion and pronation are evaluated, increased joint incongruence is physiological in hypermobile individuals and should not be confused with PLRI.
